# Modern Sociology

**Published:** 1901-12-07

**Authors:** 


### Modern Sociology.

### THE CENTRAL PUBLIC-HOUSE TRUST ASSOCIATION.

The desirableness of putting public-houses under public management has almost already ceased to be a matter for discussion. Allowing that people will drink intoxicating liquor—and the day when the British will be a teetotal nation is still far distant—public management seems to be the best method yet devised of minimising the admitted evils of liquor shops. The people who are temperate, but not teetotal, indeed see in it a possible cure for these evils altogether. The great danger of the public-house lies in the fact that it is carried on for private profit. It is as much the publican's interest to sell his customers as much whisky or beer as they can pay for as it is the grocer's to sell his patrons all the tea and sugar they can afford, or the draper's all the serge or satin. But the results are far more serious. Moreover, as the State, in its wisdom, does not allow free competition in the liquor trade, as in the grocery and drygoods, there is more chance for the average man to make money by selling liquor than by selling cloth or provisions, and thus arises a powerful "liquor party" in the community. Of the different remedies for the evils of the drink traffic, the trust system seems to be the best. The other alternatives are prohibition, which certainly does not seem to be within measurable distance of being carried out, and which, when not supported by a strong public opinion, leads to much illicit selling of intoxicants of the worst quality; restriction of licenses, which may to a slight extent limit the temptations to drink, but throws more money into the hands of those license-holders who are permitted to keep their shops, and thus does not limit or lessen the danger of creating a strong "liquor party"; and high license—selling licenses by auction to the highest bidder, which certainly limits the publican's gains, and enriches the community with the part of them, but tends rather to make the liquor-vendor try to sell as much and as cheap drink as possible, and therefore does nothing to lessen the amount of drunkenness among the community.

There remains public control by the Government, the municipality, the parish—but this is too small a unit to be safe—or the County Public House Association. Whatever be the unit chosen, the intention is to entrust the management of the public-houses of a district to people who will gain nothing by the drink sold. The persons in charge of the place will be paid a fixed salary, and if a commission is added it will be on the sale of food and non-intoxicants. The profits, beyond a moderate interest on the capital invested—4 or 5 per cent.—will be spent on some object of public utility, or on counter-attraction to the public-house; but—that the community at large may not be tempted to condone the evils of drinking because of any profit to themselves—not on saving the rates. Thus, in the mining village of Kelty, in Fife, the profits for last year went in maintaining a district nurse, at a cost of £100, and in a donation of £50 to the local library. The committee of management of this public-house have also made arrangements for the formation of a bowling-green and pavilion, at an estimated cost of £800. It is to be hoped that the founding and maintaining institutions such as the two last-named will give people something else to do in their leisure time than drink, for it must be remembered that the working men often go to the public-house because they have nowhere else to go. It is intended always to choose as the council and committee of management of these public-houses gentlemen of character and position, who will have the good of the community at heart, and who will carefully select objects on which to spend the profits which will not demoralise either the drinking or (by providing advantages which ought to be paid for out of the rates) the non-drinking class of the community.

Among the advantages of putting houses under the control of a trust may be mentioned the following:—It will not be necessary to wait until the legislature makes rules for the whole nation if in any place it is desired to carry out reforms, such as refusing to supply drink to children, abolishing sales on credit, prohibiting gambling, insisting on early closing, or entire closing on Sundays or holidays, and the non-employment of women as bar-tenders. It will be in the power, and it is within the intention, of trust companies to spend part of the profits in the buying out, where it seems desirable, of private license holders, and in some cases of shutting up the premises thus bought, so as to limit the number of public-houses in a locality.

With public-house trusts two names are notably and honorably connected—those of the Bishop of Chester, President of the People's Refreshment House Association, and Earl Grey, President of the Central Public-House Association, of which the Bishop of Chester is also a vice-president. The object of this Association is to help in the formation of trust companies in every county, to obtain licenses and manage public-houses on the lines that have been indicated. It is not itself a trust company, but an association for spreading information about trust principles, and encouraging the formation of local trust companies. Formed only in December 1900, it has been the means of organising a large number of companies, while in other places committees are at present engaged in forming companies. There are at present 17 public-house trust companies, while committees have been formed in nine districts. It is hoped that by the Brewster Sessions of 1902 there will be a trust company in every county in the three kingdoms. But if this is to be done heavy expenses must be incurred, in propagandist work, in the printing and circulating of literature bearing on the subject and in the preliminary expenses connected with the formation of companies. To meet these expenses subscriptions are asked, and should be forwarded to Mr. Oliver Williams, Secretary of the Central Public House Trust Association, 71 and 72 King William Street E.C. After November 15th, subscriptions should be sent to 114 and 116 Victoria Street, Westminster, S.W. Mr. Williams will give full information to any who wish to know more about the principles and methods of management of trust companies.

We are all temperance reformers at heart. One does not need to be exceptionally philanthropic in disposition to deplore the evils wrought by drunkenness in our midst, and to be willing to do something to diminish them. But many of us cannot fall into line with a number of temperance reformers, whose hopes seem often vain, and their methods indiscreet. But the objects of this association are eminently wholesome, and its methods practical and reasonable. A contribution to its funds may be as truly a help towards the solution of the drink problem as many a more difficult and pretentious effort. Small contributions are welcomed all the more, if those who give them add to their donation a desire to understand the working of trust companies and a readiness to spread the knowledge they gain of them. Knowledge of what has been done will surely tend to encourage the doing of more in this very practical scheme. We shall save the legislature a problem which it seems unable to solve, if we find that even with the existing licensing laws, drunkenness can be diminished, and public-houses be really places of wholesome refreshment, as they ought to be. The public-house trust offers a means of being sober without Act of Parliament.

